# Antioxidant Activity of SiO_2_@{Sericin} Hybrids: A Comparable OH-Radical and DPPH-Radical Scavenging Study

**DOI:** 10.1021/acs.langmuir.5c03085

**Published:** 2025-09-24

**Authors:** Annita Theofanous, George Theofilou, Yiannis Deligiannakis, Maria Louloudi

**Affiliations:** † Laboratory of Biomimetic Catalysis & Hybrid Materials, Department of Chemistry, University of Ioannina, Panepistimioupoli, 45110 Ioannina, Greece; ‡ Laboratory of Physical Chemistry of Materials & Environment, Department of Physics, University of Ioannina, Panepistimioupoli, 45110 Ioannina, Greece

## Abstract

A new class of hybrid materials was developed via covalent grafting of sericin, a silk-derived protein, onto SiO_2_ particles to assess their antioxidant properties. Two variants of SiO_2_@sericin hybrids were synthesized, with 10% ({SiO_2_@sericin_10}) or 20% ({SiO_2_@sericin_20}) sericin loadings. An *in tandem* analysis of their antioxidant efficiency was performed against OH and DPPH radicals. The experimental results demonstrate that the {SiO_2_@sericin} hybrids exhibit significantly enhanced antioxidant activity compared with sericin in aqueous solution. Specifically, 1 g of the {SiO_2_@sericin_10} hybrid quenches 308 μmol of DPPH radicals and 120 μmol of ^●^OH, whereas aqueous sericin in solution quenched only 85 μmol of DPPH and 53 μmol of ^●^OH. IR, Raman, BET, and DLS data collectively indicate that the interfacial topography of sericin on the SiO_2_ surface is highly dependent on its loading concentration. At the high loading (20%), sericin forms a hermetic coating over the SiO_2_ nanoparticles, resulting in steric hindrance that restricts the accessibility of antioxidant functional groups. In contrast, at the optimized 10% loading, a greater proportion of sericin’s antioxidant moieties remains accessible for radical scavenging. This interfacial topography effect is reflected in the antioxidant’s activity. One gram of {SiO_2_@sericin_20} quenches 202 μmol of DPPH radicals and 100 μmol of ^●^OH, which are smaller than the amount of {SiO_2_@sericin_10}. These findings reinforce our previous conclusion that covalent grafting of organic molecules bearing antioxidant functionalities onto SiO_2_ surfaces is an effective strategy to enhance radical scavenging efficiency, applicable to both hydroxyl-radical and DPPH (hydrogen atom transfer) quenching mechanisms.

## Graphical abstract

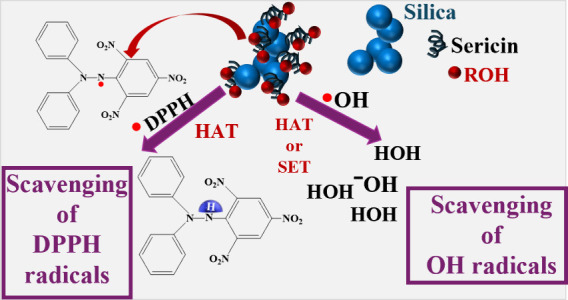

## 1 Introduction

An atom or a molecular entity carrying one or more unpaired electrons is defined as a free radical.^1^ In biotic systems, redox processes can lead to the production of free radicals,^2^ which are highly reactive and can interact with key biomolecules such as lipids, proteins, and nucleic acids. These radical interactions can cause structural and functional modifications to the targeted molecules, ultimately resulting in widespread tissue dysfunction and injury.^3^

Of particular interest are hydroxyl radicals (^●^OH),^4^ which are characterized by an extremely short half-life on the order of 10^–10^ s.^5,6^ In addition, due to their exceptionally high one-electron reduction potential (1.8–2.7 V vs NHE),^2,7^ hydroxyl radicals are highly reactive, being able to cause significant damage to host cells by inducing oxidative modifications of amino acids.^8^ A powerful strategy to mitigate these harmful effects is the use of antioxidants.^9^ Antioxidants exert their protective effects by interacting with free radicals, neutralizing them, and stopping the spread of harmful chain reactions, mainly through electron or hydrogen atom donation.^10,11^

Sericin, the silk protein that contributes to the structural integrity of silk cocoons,^12^ is routinely extracted in significant quantities as a byproduct of the silk-textile industry^13,14^ and can exhibit antioxidant capacity, neutralizing free radicals that are implicated in oxidative damage.^15,16^ Sericin has a molecular weight of 24–400 kDa, composed of 18 distinct amino acids, with mostly polar side chains, i.e., serine, histidine, glycine, threonine, tyrosine, aspartic acid, and glutamic acid being the most prevalent.^17,18^ The antioxidant potential of sericin, which is used as a natural preservative in the food industry, is attributed to its high content of amino acids with hydroxyl groups, predominantly serine.^17^ The presence of phenolic and flavonoid compounds within the adjacent layers of the sericin protein may also enhance the antioxidant activity of sericin.^19,20^

Employing modification, precipitation, or cross-linking with other polymers, sericin can be developed into stable and controllable biomaterials.^21,22^ Das et al. extracted raw sericin from the silk cocoons of *Bombyx mori* and utilized it as a reducing agent to synthesize gold nanoparticles.^23^ Curcumin-loaded sericin nanoparticles (Cur-SNPs) were prepared by cross-linking with genipin (Gn), and their antioxidant activity was studied in vivo in rats.^24^ Sericin nanoparticles (SNs) can be synthesized and integrated into nanocomposite films to improve their barrier properties.^25,26^ The antioxidant activity of the films was assessed using 2,2-diphenyl-1-picrylhydrazyl (DPPH),^25^ which is a stable molecule containing a nitrogen-based free radical.^27^ Sericin nanoparticles were also synthesized using crocetin as a novel bioactive natural cross-linker (NPc) and compared to sericin nanoparticles cross-linked with glutaraldehyde (NPg); they were assessed using the DPPH assay.^28^ SS-MgO nanoparticles were synthesized by conjugating sericin protein with magnesium oxide nanoparticles^29^ and silver nanoparticles (S-AgNPs).^30^ Orlandi et al. developed sericin-based nanoparticles encapsulating natural polyphenols, including proanthocyanidins (P), quercetin (Q), and epigallocatechin gallate (EGCG).^31^ Their antioxidant activity was evaluated using the DPPH assay.^29–31^

Within the advent of hybrid materials, silica can serve as a versatile matrix for the development of novel hybrid materials, owing to its highly adaptable surface.^32^ The immobilization of organic groups on the SiO_2_ surface can be achieved through established chemical modification techniques.^33–36^ With regard to antioxidant hybrids, Catauro et al. synthesized silica-based hybrid materials incorporating poly­(ε-caprolactone) through the sol–gel process; their antioxidant activity was evaluated using the DPPH and 2,2′-azino-bis­(3-ethylbenzothiazoline-6-sulfonic acid) (ABTS) assays.^37^ Grafting of humic acid moieties onto the silica surface resulted in a hybrid humic acid-SiO_2_ nanomaterial, assessed by the DPPH assay.^38^ MnS_2_-SiO_2_,^39^ chitosan-silica (CS-Si),^40^ and SiO_2_/caffeic acid hybrid materials^41^ were also synthesized and evaluated as antioxidants by the DPPH and/or ABTS methods. The hybrid nanoantioxidant MSN-morin was prepared by the immobilization of morin (2′,3,4′,5,7-pentahydroxyflavone) on mesoporous silica (MSN) nanoparticles and assessed using electron paramagnetic resonance (EPR) spectroscopy to quantify its ability to neutralize hydroxyl radicals (HO^●^).^42^

In our previous works, we have shown that gallic acid (GA) immobilized on SiO_2_ shows considerable antioxidant activity against DPPH radicals^43^ or OH radicals.^44^ In addition, we have shown that components of hyaluronic acid, i.e., d-flucuronic acid (GLA) and *N*-acetyl-d-glucosamine (GLAM), immobilized on SiO_2_, resulted in novel hybrid materials SiO_2_@GLA and SiO_2_@GLAM,^43,45^ respectively, that exhibited antioxidant activity via the hydrogen atom transfer (HAT) mechanism^43,45^ and OH quenching antioxidant activity.^44^ In all cases,^43–45^ the SiO_2_ matrix was shown to play an active role in fine-tuning of the antioxidant activity, i.e., via surface–OH and H-bonding contributions. The beneficial role of the SiO_2_ matrix was also verified in more complex systems, e.g., polyphenolic polymer materials [humic acid-like polycondensate (HALP)] on SiO_2_ nanoparticles that demonstrated improved antioxidant activity against DPPH radicals.^46^

In this context, herein, our key hypothesis was that SiO_2_ would affect the antioxidant properties of sericin grafted onto its surface. To address this, we have synthesized two {SiO_2_@sericin} hybrids with different sericin loadings (10% or 20%), {SiO_2_@sericin_10} or {SiO_2_@sericin_20}, respectively, using silica as the matrix and sericin protein as the antioxidant component (Scheme 1). The hybrid materials were assessed for their capacity to scavenge DPPH as well as OH radicals. As we have stressed recently,^44,45^ *in tandem* the DPPH and OH antioxidant assay provides a more complete understanding of the underlying physicochemical mechanisms involved in the antioxidant activity of a given antioxidant.^44,45^ Here we underline that the two assays are performed via different experimental protocols. The DPPH-radical scavenging protocol,^43,45,47,48^ which is usually carried out by UV–vis spectrophotometry, provides a well-established method for assessing the HAT capacity. However, the investigation of highly reactive hydroxyl radicals presents greater analytical challenges due to their short-lived nature. Effective detection typically requires the use of spin-trapping techniques combined with electron paramagnetic resonance (EPR) spectroscopy.^44,49–52^ Controlled generation of •OH radicals can be achieved via the Fenton reaction, with subsequent detection and quantification performed through EPR analysis.^50–52^

**Scheme 1:**
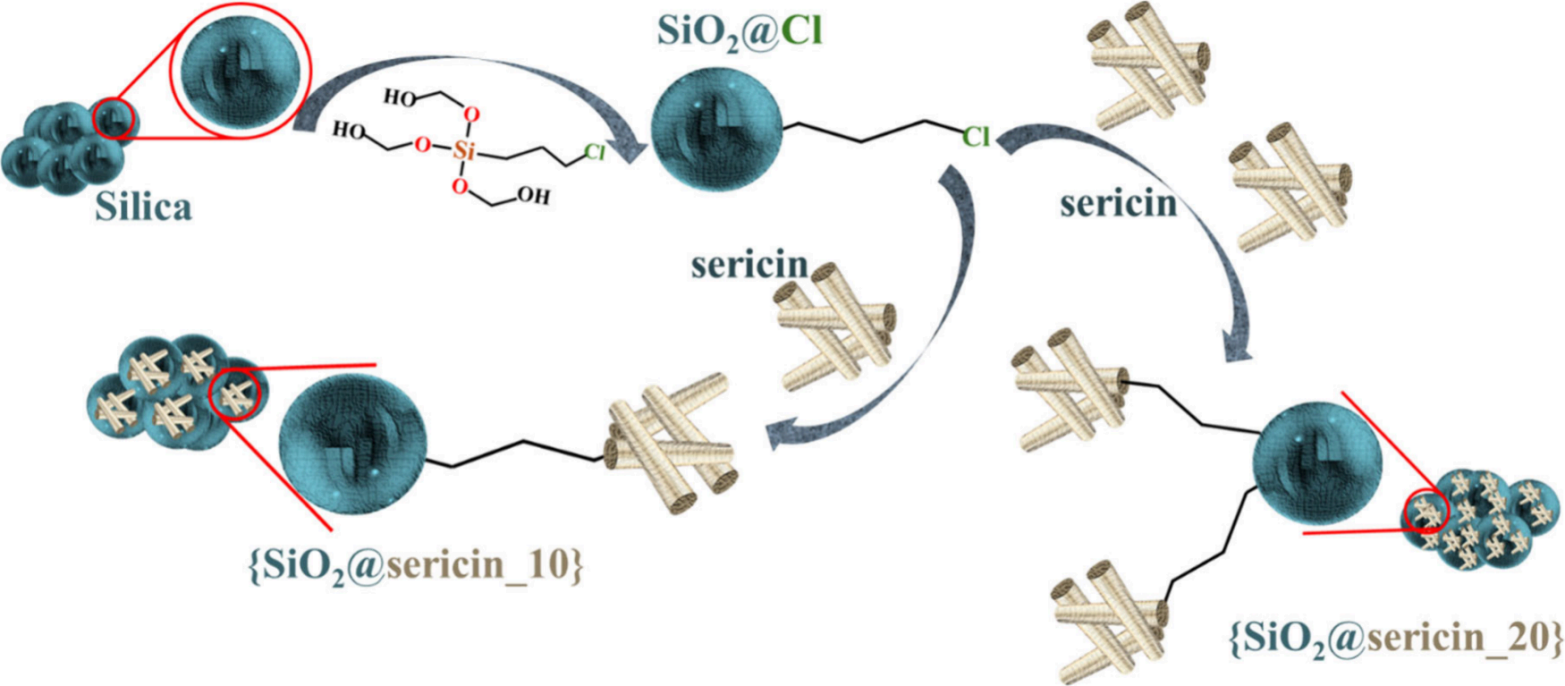
Schematic Illustration of the Synthesis of the Hybrid Materials {SiO_2_@sericin_10} and {SiO_2_@sericin_20}

The objectives of the present study were (a) to synthesize {SiO_2_@sericin} hybrid materials by immobilizing sericin onto the silica surface (Scheme 1), (b) to understand the interfacial properties of the hybrid materials using TG-DTA, dynamic light scattering, BET-porosimetry, FT-IR, and Raman spectroscopy, and (c) to perform a comparative evaluation of the antioxidant capacity of the materials against OH and DPPH radicals.

## 2 Materials and Methods

### 2.1 Chemicals and Sericin

Sericin (aqueous solution from *B. mori*) was purchased from DSM Nutritional Products Ltd. (Kaiseraugst, Switzerland). 3-Chroropropyl-trimethoxysilane (>99% purity) was obtained from Sigma-Aldrich. SiO_2_ (SSA = 340 m^2^/g) was obtained from Merck. Solvents, including acetone (>99.8% purity) and methanol (>99.9% purity), were purchased from Merck. 2,2-Diphenyl-1-picrylhydrazyl (DPPH) was procured from Sigma-Aldrich. Ferrous­(II) sulfate heptahydrate (FeSO_4_·7H_2_O, >99.5%) was acquired from Fluka, while a hydrogen peroxide solution (H_2_O_2_, 30% (w/w)) and 5,5-dimethyl-1-pyrroline *N*-oxide (DMPO) were obtained from Sigma-Aldrich. Chromatography-grade water (LC-MS grade, 2.5 L) was obtained from Merck.

### 2.2 Synthesis of Hybrid Material SiO_2_@sericin (10% sericin loading)

First, 2.78 g of SiO_2_ that had been dried at 140 °C for 24 h was dispersed in 15 mL of methanol, and the obtained slurry solution was subjected to ultrasonic agitation for 5 min. Subsequently, 2.5 mL of (3-chloropropyl)­trimethoxysilane was added to the mixture, which was refluxed for 24 h at 60 °C. The recovered chloropropyl-functionalized SiO_2_ material, denoted SiO_2_@Cl, was rinsed with methanol and acetone and allowed to dry in a Dry-Pistol for 24 h at 40 °C. Then, 1.6 g of SiO_2_@Cl was dispersed in 17 mL of a sericin solution. The slurry solution was transferred to a stirring bath under reflux and maintained at 80–85 °C for 24 h. Following this period, hybrid material {SiO_2_@sericin_10} obtained by filtration was rinsed with methanol and acetone and allowed to dry under vacuum for 24 h at 40 °C.

### 2.3 Synthesis of Hybrid Material SiO_2_@sericin (with 20% sericin loading)

Hybrid material SiO_2_@sericin (20%) was synthesized using an analogous methodology with slight protocol alterations, as follows. First, 1.25 g of dried SiO_2_ was added in 20 mL of methanol, and the mixture was exposed to ultrasonic agitation for 5 min to ensure thorough mixing and dispersion followed by the addition of 1 mL of (3-chloropropyl)­trimethoxysilane. The mixture was stirred under reflux at 60 °C for 24 h. The obtained SiO_2_@Cl was rinsed using methanol and acetone and dried under vacuum for 24 h at 40 °C. Then, a 0.5 g portion of the dried SiO_2_@Cl was dispersed in 21 mL of a sericin solution, and the mixture was stirred under reflux at 80–85 °C for 48 h. The synthesized {SiO_2_@sericin_20} material was rinsed with methanol and acetone and dried under vacuum at 40 °C for 24 h.

Table 1 lists all of the key characteristics of the materials. In each synthesis, before the grafting of sericin, precursor material SiO_2_@Cl was synthesized, i.e., where the chlorosilane was grafted (see Figure 1). Thus, for sericin loadings of 10% and 20% listed in Table 1, precursors SiO_2_@Cl_10 and SiO_2_@Cl_20, respectively, were used.

**Table 1:** Morphological Characteristics of SiO_2_ Hybrid Materials

hybrid material	loading (%)	BET	DLS aggregate size (nm)	point of zero charge (pH)
{SiO_2_@sericin_10}	10	310	850 ± 60	3.44
{SiO_2_@sericin_20}	20	211	550 ± 40	3.76

**Figure 1:**
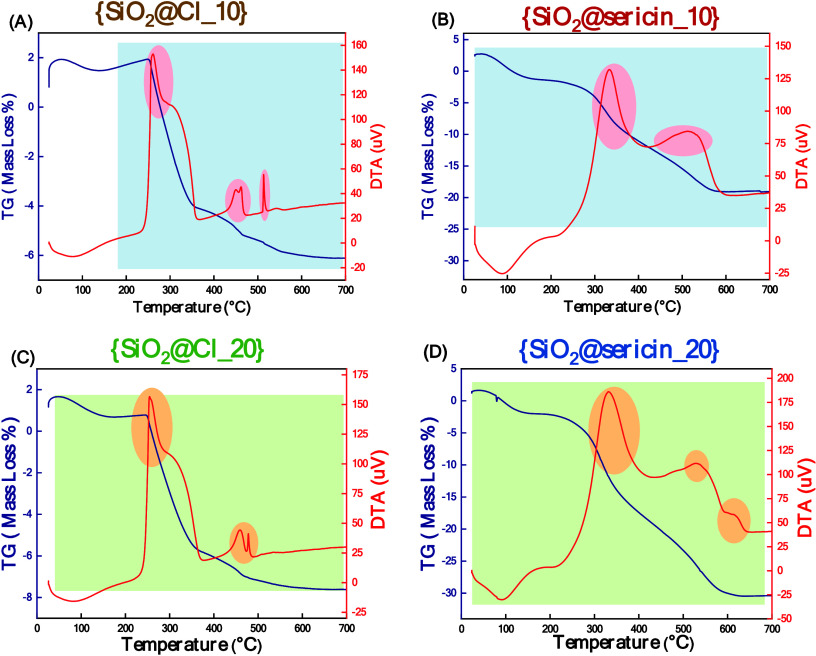
Thermogravimetric (TG) and differential thermal analysis (DTA) were performed on the following samples: (A) {SiO_2_@Cl_10}, (B) {SiO_2_@sericin_10}, (C) {SiO_2_@Cl_20}, and (D) {SiO_2_@sericin_20}. Thermal analysis of all materials was performed at temperatures from 20 to 700 °C at a rate of 10 °C/min, under synthetic air flow.

As detailed in this study, the 10% and 20% sericin loadings were selected based on preliminary screening experiments to represent two distinct interfacial coverage regimes. The 10% loading in the {SiO_2_@sericin_10} hybrid corresponds to moderate surface functionalization, wherein portions of the SiO_2_ surface remain exposed. In contrast, the 20% loading in the {SiO_2_@sericin_20} hybrid results in nearly complete surface coverage by sericin, representing a regime of maximal grafting density.

It should be emphasized that the designations SiO_2_@sericin_10 and SiO_2_@sericin_20 are defined by the final organic loadings determined by TGA (∼10 and ∼20 wt %, respectively), rather than by the concentrations of the precursor solutions. Both hybrids were prepared from sericin solutions of identical concentration (100 ppm), but the syntheses differed in the amounts of SiO_2_@Cl employed and in the reaction times (24 h vs 48 h). These factors governed the extent of incorporation of sericin into the hybrid, leading to the distinct loading regimes. The detailed preparation conditions and corresponding organic loadings are summarized in Table 2.

**Table 2:** Initial Synthesis Conditions and Final Sericin Loadings of the SiO_2_@sericin Hybrids

hybrid material	SiO_2_@Cl (g)	sericin volume (mL)	sericin concentration (ppm)	reaction time (h)	loading (%) (TG/DTA)
{SiO_2_@sericin_10}	1.6	17	100	24	10
{SiO_2_@sericin_20}	0.5	21	100	48	20

### 2.4 Instrumentation

#### 2.4.1 BET Specific Surface Areas (SSAs)

The specific surface areas (SSAs) of the samples were determined by using N_2_ adsorption/desorption isotherms. These isotherms were measured at 77 K using a Quantachrome NOVAtouch LX2 instrument. Before measurements, the samples were degassed under vacuum at 150 °C for 16 h. The SSA was calculated using the Brunauer–Emmett–Teller (BET) method, based on the adsorption and desorption data points. The specific surface area (SBET) was determined using adsorption data points within the relative pressure (*P*/*P*_0_) range of 0.1–0.3. The pore radius was calculated using the Barrett–Joyner–Halenda (BJH) method based on the adsorption data points at *P*/*P*_0_ values from 0.35 to 0.99. The total pore volume was obtained at a *P*/*P*_0_ values of 0.99.

#### 2.4.2 Thermogravimetric Analysis (TG-DTA)

The loading capacity of modified materials was quantified using a Setaram TG-DTA (thermogravimetric-differential thermal analysis) instrument. For the analysis of the SiO_2_@Cl and SiO_2_@sericin hybrid materials, approximately 40 mg of material was used. Thermal analysis was performed over the temperature range of 20–700 °C, with a heating rate of 10 °C/min, under a controlled flow of synthetic air.

#### 2.4.3 Fourier Transform Infrared Spectroscopy (FT-IR)

FT-IR measurements were performed by using a Nicolet IS-5 FT-IR spectrometer. The samples were prepared as potassium bromide (KBr) pellets, with SiO_2_ particles obtained from various SiO_2_@Cl and SiO_2_@sericin samples mixed with KBr and subjected to uniaxial compression (10 N) using a hydraulic press. This process produced compressed tablets with a uniform diameter of 1 cm and variable heights ranging from 1 to 15 mm. The final spectra represent an average of 32 individual scans, acquired over the spectral range of 400–4000 cm^–1^ with a resolution of 2 cm^–1^.

#### 2.4.4 Raman Spectroscopy

Raman spectra were recorded over 10 s, integrating data from 50 scans to achieve a high signal-to-noise ratio. The measurements were performed by using a HORIBA Xplora Plus spectrometer, which was interfaced with an Olympus BX41 microscope and utilized a 785 nm diode laser for excitation.

#### 2.4.5 Electron Paramagnetic Resonance (EPR) Spectroscopy

EPR spectra were recorded using a continuous-wave Bruker ER200D spectrometer, which was paired with an Agilent 5310A frequency counter for accurate frequency measurements. The operation of the spectrophotometer was controlled by using custom software developed on the LabView platform. The parameters for EPR spectrum acquisition were set as follows: modulation amplitude of 10 G from peak to peak (pp), modulation frequency of 100 kHz, and microwave power of 20 mW. The spectra were recorded at room temperature under ambient conditions.

#### 2.4.6 UV–Vis Spectroscopy

A Hitachi model U-2900 spectrophotometer, equipped with a Unisoku cryostat integrated into the UV–vis spectrophotometer beam chamber, was utilized for the measurements. Consequently, the system provided precise digital control of the sample temperature over the range of 100 to −100 °C, with a temperature stability of ±0.1 °C. Sample cooling was achieved using a regulated flow of cold nitrogen (N_2_) gas, generated from the vaporization of liquid nitrogen (N_2_) coolant.

#### 2.4.7 Dynamic Light Scattering (DLS)

A Horiba Nano Particle Analyzer SZ-100 V2, 10 mW, 532 nm instrument was used to determine the ζ potential (ZP) and the particle size (PS) of the materials in aqueous solution. For particle size measurement, the laser light can be scattered by the sample particles at either 90° or 173° and is collected through a lens and a pinhole by the detector. The 90° geometry was chosen for our samples because its scattered light intensity was strong enough to be detected while still providing good sensitivity to particle motion.

For ζ potential measurements, laser light is divided into two beams: a reference beam and a scattered light beam. The scattering angle is 173° as it captures sufficient scattered intensity while reducing unwanted background noise. The reference beam undergoes modulation^53^ and interferes with the scattered beam in a prism, and the resultant waveform is then changed to a digital signal to be calculated. For complete mapping of the ZP versus pH, a Horiba pH controller (model LY-711) was used to automatically adjust the pH using 0.1 M HNO_3_ and 0.1 M NaOH. The particles were circulated through the measuring flow cell using a peristaltic pump.

For ζ potential measurements, we performed titrations from acidic to basic pH values. Specifically, we tested pH values from 2 to 9 using 0.1 M HNO_3_ and 0.1 M NaOH to adjust the pH. Each of the samples was mildly sonicated for 15 min (20 W sonication bath) at room temperature and then dispersed in 100 mL of deionized H_2_O. The measured samples were as follows: SiO_2_, sericin, SiO_2_@sericin_10, and SiO_2_@sericin_20. The sample masses used for each titration were 30, 6, 35, and 35 mg, respectively. Each dispersion, after mild sonication, was allowed to equilibrate at the starting pH value for 15 min under mild stirring in the DLS titrator, and then pH titration was performed.

### 2.5 Evaluation of Antioxidant Radical Scavenging Capacity (RSC)

The HAT antioxidant activity of {SiO_2_@sericin_20} and {SiO_2_@sericin_10} was evaluated using the DPPH-radical assay. This method allows the HAT reaction kinetics to be monitored via the 515 nm (A_515_) absorbance of the DPPH radical.^54^ Quantitation of the DPPH-radical concentration in a methanol solution is carried out following the standardized protocol established by Brand-Williams et al.^55^ Quantitatively, the quenched DPPH radicals are reliably estimated by the Lambert–Beer law^56^ *A* = ε*c* *l*, where *A* represents the absorbance at 515 nm, ε denotes the molar extinction coefficient of DPPH in methanol (ε = 1.09 × 10^4^),^43^ *c* represents the concentration of DPPH, and *l* denotes the path length of the sample, which is 1 cm.

### 2.6 Assessment of the Hydroxyl-Radical Scavenging Capacity

The hydroxyl-radical scavenging capacity (OH-RSC) of the hybrid materials was evaluated by using EPR spectroscopy. ^●^OH radicals were generated through the Fenton reaction.^51^ The methodology employed for the generation of a controllable amount of ^●^OH has been comprehensively detailed in our previous study.^44^

The generated ^●^OH radicals were trapped using a 6.67 mM solution of DMPO (5,5-dimethyl-1-pyrroline *N*-oxide).^50^ In a total reaction volume of 3 mL, 20 μL of the DMPO-OH stock suspension was dispensed into glass capillaries. These capillaries were then subjected to in situ irradiation within the EPR cavity to measure the EPR signals. Quantitative analysis of hydroxyl radicals was carried out using DPPH (1,1-diphenyl-2-picrylhydrazyl) as a spin standard^50,51^ using the double integral of EPR signals.^44,50^

## 3 Results and Discussion

### 3.1 Characterization of SiO_2_@sericin Hybrid Materials

#### 3.1.1 Thermogravimetric Analysis

Figure 1 shows the thermogravimetric data of hybrid materials {SiO_2_@sericin_10} and {SiO_2_@sericin_20}. For the sake of completeness, we also present the TGA data for {SiO_2_@Cl _10} and {SiO_2_@Cl _20}. In Figure 1, the red lines represent the exothermic and endothermic transitions observed in differential thermal analysis (DTA), while the blue lines (TG) illustrate the cumulative mass-loss profile of the materials under study.

According to Figure 1A, in the case of {SiO_2_@Cl_10} the total mass loss from 199 to 700 °C was Δ*m*_total_SiO_2_@Cl_10 = 7.8% corresponding to 1.1 mmol of Cl­(CH_2_)_3_/g of {SiO_2_@Cl_10}. An exothermic change also occurs in the range of 250–350 °C, due to the combustion of the organic. Thermal analysis of {SiO_2_@sericin_10} (Figure 1B) shows a total mass loss from 200 to 700 °C and Δ*m*_total_SiO_2_@sericin_10 = 17.6%. Since {SiO_2_@sericin_10} is derived from {SiO_2_@Cl_10}, the net organic loss is Δ*m*_net_{SiO_2_@sericin_10} = Δ*m*_total_{SiO_2_@sericin_10} – Δ*m*_total_SiO_2_@Cl_10 = 17.6% – 7.8% = 9.8%, which corresponds to 97.4 mg of sericin/g of {SiO_2_@sericin_10} (listed in Table 1). The first mass-loss event (∼200–300 °C) is attributed to decomposition of low-molecular weight fragments and sericin side chains, while the second one (∼320–380 °C) corresponds to the degradation of the protein backbone (amide linkages).

Figure 1C displays the thermogravimetric analysis of {SiO_2_@Cl_20}, used as a precursor for hybrid material {SiO_2_@sericin_20}. The cumulative mass loss was Δ*m*_total_SiO_2_@Cl_20 = 8.3%, corresponding to 1.2 mmol of Cl­(CH_2_)_3_/g of {SiO_2_@Cl_20}. The TG for {SiO_2_@sericin_20} (Figure 1D) shows a mass loss Δ*m*_total_SiO_2_@sericin_20 = 28.2%; thus, Δ*m*_net_SiO_2_@sericin_20 = Δ*m*_total_SiO_2_@sericin_20 – Δ*m*_total_SiO_2_@Cl_20 = 8.2% – 8.3% = 19.9%, which corresponds to 198.9 mg of sericin/g of {SiO_2_@sericin_20}. The thermal profile compared to that of {SiO_2_@sericin_10} shows additionally a third, broader, and less intense mass-loss feature at ∼400–500 °C, which is assigned to more tightly bound sericin domains strongly interacting with the silica surface. This additional high-temperature event, along with the earlier decomposition steps, reflects the increased grafting density and multilayer coverage of sericin on SiO_2_@sericin_20 compared to SiO_2_@sericin_10.

Overall, the present TGA data show that our synthetic protocols allow controlled grafting of the sericin on the SiO_2_ particles. From the loading data, as listed in Table 1, the {SiO_2_@sericin_10} hybrid contained 97.4 mg of sericin/g while {SiO_2_@sericin_20} contained 198.9 mg of sericin/g.

#### 3.1.2 Vibrational Spectroscopy Analysis (FT-IR and Raman)

##### 3.1.2.1 FT-IR Spectroscopy

Figure 2A presents the FT-IR spectra for {SiO_2_@sericin_10} and {SiO_2_@Cl_10}, alongside spectra for nonfunctionalized SiO_2_ and sericin. The FT-IR for {SiO_2_@Cl_20} (not shown) was similar to that for {SiO_2_@Cl_10}. The FT-IR spectrum of SiO_2_ (Figure 2A (iii)) is characterized by peaks at 467, 815, and 1089 cm^–1^, corresponding to the rocking vibrations (R), symmetric stretching vibrations (SS), and asymmetric stretching vibrations (AS), respectively, of the Si–O–Si bond within the silica network.

**Figure 2:**
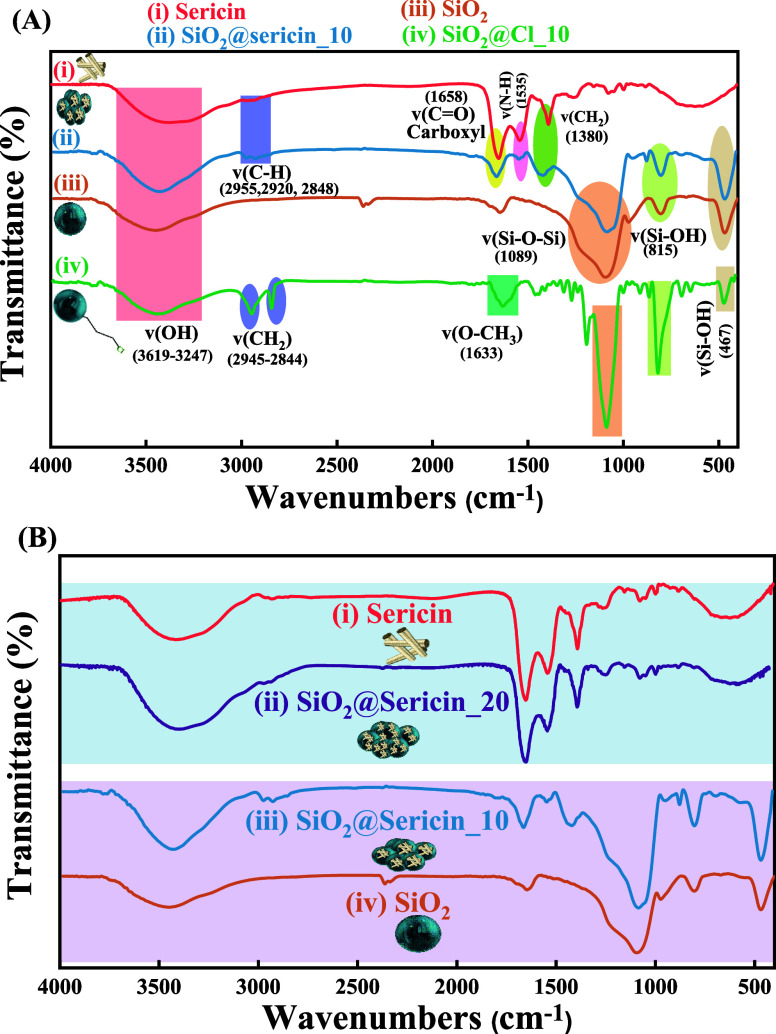
Fourier transform infrared (FT-IR) spectra for (A) sericin (i), hybrid material SiO_2_@sericin_10 (ii), SiO_2_ (iii), and SiO_2_@Cl_10 (iv) and (B) sericin (i), hybrid material SiO_2_@sericin_20 (ii), hybrid material SiO_2_@sericin_10 (iii), and SiO_2_ (iv).

In the FT-IR spectrum of {SiO_2_@Cl_10} (Figure 2A (iv) (green line)), the presence of characteristic peaks from both the organosilane and SiO_2_ confirms the successful immobilization of the organosilane onto the silica surface. A broad flattened peak observed in the range of 3659–3213 cm^–1^ corresponds to the stretching vibration of the O–H bond. The peaks observed at 2945 and 2844 cm^–1^ are attributed to the asymmetric stretching vibrations of the ν­(C–H) bonds.^57^ The peak observed at 1633 cm^–1^ indicates the presence of the ν­(O–CH_3_) bond. Finally, the peaks observed at 467, 815, and 1089 cm^–1^ correspond to the characteristic symmetric stretching vibrations (SS) and asymmetric stretching vibrations (AS) of the Si–O–Si bond, respectively.^58,59^

The FT-IR spectrum of the sericin protein is presented in Figure 2A (i). The broad peak observed at 3420 cm^–1^ is attributed to the stretching vibrations of the ν­(OH) bonds. The peaks at 2974, 2928, and 2854 cm^–1^ are attributed to the stretching vibrations of the ν­(C–H) bonds of the sericin proteinic fragments.^54^ Additionally, the peak at 1658 cm^–1^ is attributed to the stretching vibrations of the ν­(C=O) bonds.^36,54^ The peaks observed at 1640, 1438, and 1238 cm^–1^ are attributed to distinct amide bond vibrations. That at 1640 cm^–1^ corresponds to amide I, which is associated with ν­(C=O) stretching.That at 1438 cm^–1^ is attributed to amide II, encompassing δ­(N–H) deformation and ν­(C–N) stretching.That at 1238 cm^–1^ is linked to amide III, involving ν­(C–N) stretching and δ­(N–H) deformation.^36,54^

In Figure 2A (ii), the FT-IR spectrum evidences the successful immobilization of the sericin protein onto the silica surface, i.e., by the presence of characteristic peaks corresponding to both sericin and silica. The broad peak observed at 3420 cm^–1^ is attributed to the stretching vibrations of the ν­(OH) bonds. The peaks observed at 2974, 2928, and 2854 cm^–1^ correspond to the stretching vibrations of the ν­(C–H) bonds in the sericin protein.^54^ Additionally, the peak observed at 1658 cm^–1^ is attributed to the stretching vibrations of the ν­(C=O) bonds, which are characteristic of the amide I band in the β-sheet structure.^36,54^ The peaks observed at 1658, 1535, and 1380 cm^–1^ correspond to the formation of amide bonds, specifically amide I (ν­(C=O) stretching), amide II (δ­(N–H) deformation and ν­(C–N) stretching), and amide III (ν­(C–N) stretching and δ­(N–H) deformation), respectively. These appear displaced relative to natural silk, which contains both sericin and fibroin, due to the disordering of sericin in the spatial arrangement. The peaks observed at 1640, 1438, and 1238 cm^–1^ correspond to the formation of amide bonds, specifically amide I (ν­(C=O) stretching), amide II (δ­(N–H) deformation and ν­(C–N) stretching), and amide III (ν­(C–N) stretching and δ­(N–H) deformation), respectively.^54^ Finally, we see all three peaks due to silica at 467, 815, and 1089 cm^–1^ correspond to the characteristic symmetric stretching vibrations (SS) and asymmetric stretching vibrations (AS) of the Si–O–Si bond, respectively.^58,59^

As illustrated in Figure 2B (ii), the FT-IR spectrum of the SiO_2_@sericin_20 hybrid material is practically identical with that of the aqueous sericin solution. This indicates that at such high sericin loadings on the silica surface, i.e., 194 mg of sericin/g, the FT-IR spectrum is dominated by those of sericin while the SiO_2_ peaks diminish or become undetectable. We point out the characteristic difference of the FT-IR spectrum of SiO_2_@sericin_10 (Figure 2B (iii)), where it exhibits characteristic peaks corresponding to both sericin and SiO_2_. Thus, the FT-IR spectra provide a structural hint. At 20% sericin loading on the silica surface, the sericin protein overwhelmingly covers the SiO_2_ surface, while at 10% sericin loading, the sericin protein partially covers the SiO_2_ surface. As we show hereafter, these differences in the interfacial coating have a discrete effect on the physicochemical properties and the radical scavenging properties for the two hybrids.

##### 3.1.2.2 Raman Spectroscopy

Figure 3 presents the Raman spectra of SiO_2_, SiO_2_@Cl_10, and hybrid SiO_2_@sericin_10. The vibrational modes of the SiO_2_ matrix, specifically the δ­(Si–O–Si) breathing modes, are detected in the range of 300–600 cm^–1^.^60^ The band at 480 cm^–1^ is associated with the characteristic formation of four-membered siloxane rings (4MRs).^51,61^ Furthermore, the silica matrix displays a characteristic band at 981 cm^–1^, corresponding to symmetric stretching vibration ν­(Si–O–Si).^60^ The peak at 796 cm^–1^ indicates the existence of the ν­(Si–O–H) vibration.^45,61^

**Figure 3:**
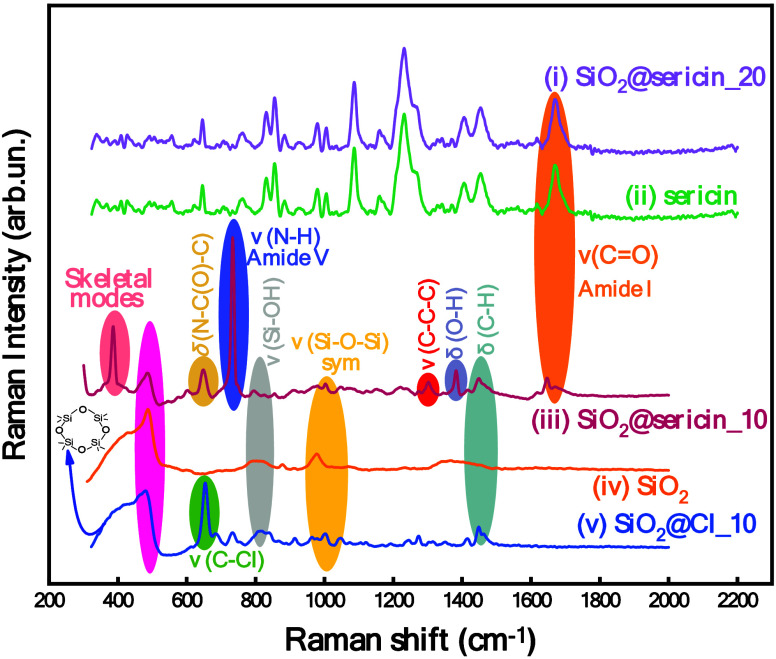
Raman spectra of (i) hybrid material SiO_2_@sericin_20, (ii) sericin, (iii) hybrid material SiO_2_@sericin_10, (iv) SiO_2_, and (v) SiO_2_@Cl_10.

The Raman spectrum of the SiO_2_@Cl_10 material (Figure 3(v)) exhibits an initial four-membered siloxane ring (4MRs) peak at 480 cm^–1^.^51,61^ The peak at 657 cm^–1^ is assigned to the ν­(C–Cl)^57^ bond. Subsequently, at 796 and 981 cm^–1^, two distinct peaks are observed, corresponding to the ν­(Si–O–H)^45,61^ and ν­(Si–O–Si) vibrations,^60^ respectively. Finally, the peak observed at 1450 cm^–1^ is attributed to the δ­(C–H) bending vibration, assigned to the propyl carbon chain.^57^

The Raman spectrum of free sericin is presented in Figure 3 (ii). The peaks observed at 1674 cm^–1^ correspond to the stretching vibrations of the ν­(C=O) bond,^57^ indicating the presence of the amide I group.^57^ Additionally, the characteristic peaks in the range of 1300–1270 cm^–1^ associated with amide III, which define the α-helix structure, are absent from this spectrum. This absence is attributed to the spatial disorder of sericin, indicating a lack of well-defined secondary structure.^57^ The peak observed at 1087 cm^–1^ is attributed to the ν­(C–O) vibration associated with CH_2_OH bonds.^57^ Furthermore, the peaks in the range of 1008–980 cm^–1^ correspond to ν­(C–C) bond vibrations, while those in the region of 900–800 cm^–1^ indicate the presence of symmetric ν­(CNC) vibrations.^54,62^ The band observed at 764 cm^–1^ is attributed to the amide V ν­(N–H) bond vibration.^57^

The Raman spectrum of the SiO_2_@sericin_10 material (Figure 3 (iii)) contains the band at 480 cm^–1^, attributed to SiO_2_, i.e., the characteristic formation of four-membered siloxane rings (4MRs).^57,63^ The band at 734 cm^–1^ is assigned to the amide V vibration, specifically the ν­(N–H) stretch.^54,57^ The peaks at 796 and 981 cm^–1^ correspond to the ν­(Si–O–H)^45,61^ and ν­(Si–O–Si) vibrations,^60^ respectively. The peak appearing at 1302 cm^–1^ indicates the existence of the ν­(C–C–C) vibration.^54^ Additionally, peaks associated with the δ­(O–H) and δ­(C–H) bending vibrations are observed at 1377 and 1451 cm^–1^, respectively.^57^ Finally, the peak observed at 1642 cm^–1^ is attributed to the ν­(C=O) stretching vibration, which is characteristic of the amide I band.^54^

Interestingly, the Raman spectrum of SiO_2_@sericin_20 (Figure 3 (i)) is identical to that of sericin, indicating that at 20% loading the sericin layer has entirely covered the SiO_2_ surface; i.e., a similar phenomenon is observed in the FT-IR spectra (Figure 2B). At the lower loading, i.e., 10%, the Raman spectrum of SiO_2_@sericin_10 exhibits spectral features of both sericin and the SiO_2_ matrix (Figure 3 (ii–iv)).

Overall, the present Raman data (i) confirm the successful immobilization of sericin onto the SiO_2_ surface (ii). The 10% loading provides an interface where the SiO_2_ surface is not fully covered by the grafted sericin; on the contrary, the 20% loading fully covers the SiO_2_ surface. This information allows us to anticipate two distinct types of interfacial topography. SiO_2_@sericin_10 consists of sericin “islands” partially covering SiO_2_, allowing interfacial reactivity between the liquid medium and both the sericin and the silica surface. In contrast, SiO_2_@sericin_20 consists of a thick layer of sericin surrounding the SiO_2_, and thus, the interfacial dynamics is preferably determined by the sericin only.

#### 3.1.3 Interfacial Topography Materials

The surface functionalization of SiO_2_ particles is evidenced through observable color changes (see Figure 4A1–A). The originally white SiO_2_ (Figure 4A1) becomes white-yellow as SiO_2_@sericin_10 (Figure 4A2) and faint yellow for SiO_2_@sericin_20 (Figure 4A3). The evaluation of the specific surface area (SSA) of the materials provides critical insights into the interfacial topography, as postulated by IR and Raman also. Specifically, as shown by their N_2_ adsorption–desorption isotherms, all three materials exhibit type IV isotherms,^61^ characteristic of mesoporous structures. The estimated SSA values (see bar graphs in Figure 4B) obtained by BET analysis^61^ of the data reveal that surface grafting results in a decrease in SSA from 414 m^2^/g for SiO_2_ particles to 310 m^2^/g for SiO_2_@sericin_10 and 211 m^2^/g in SiO_2_@sericin_20. Thus, immobilization of sericin at 20% on the silica surface results in a 50% decrease in the material’s specific surface area compared to unmodified silica. A similar trend is observed in pore volume and pore radius (Figure 4C and Figure S1), where an increase in organic loading on the silica surface results in a corresponding decrease in both parameters.

**Figure 4:**
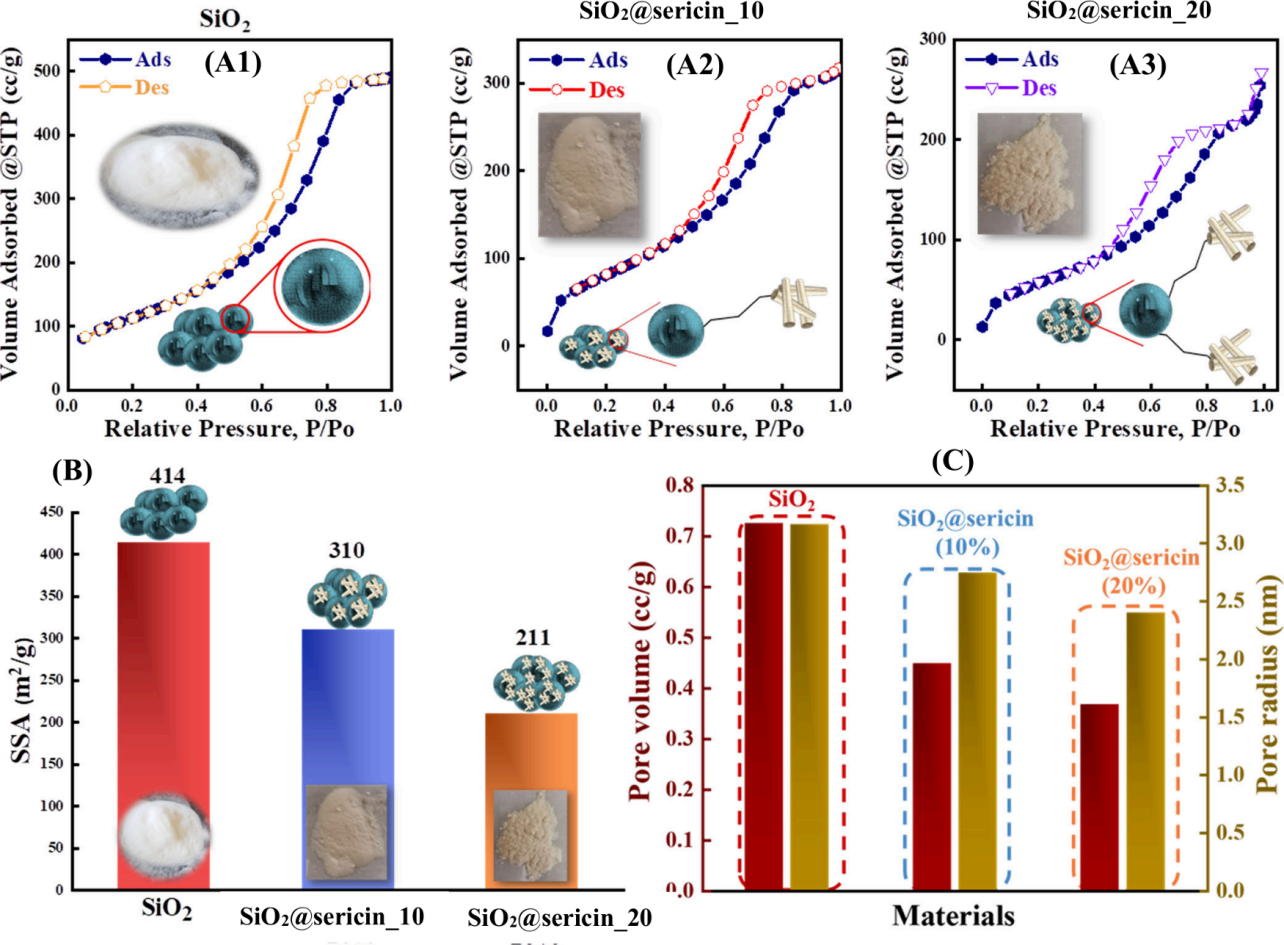
(A) N_2_ adsorption–desorption isotherms for SiO_2_ (A1), SiO_2_@sericin_10 (A2), and SiO_2_@sericin_20 (A3). (B) Specific surface area (SSA) values for SiO_2_, SiO_2_@sericin_10, and SiO_2_@sericin_20, obtained from the BET analysis of the isotherms in panel A. (C) Pore volumes and pore radii of the materials.

Overall, the present SSA and pore analysis corroborate our IR and Raman data, confirming that the interfacial topography of SiO_2_, SiO_2_@sericin_10, and SiO_2_@sericin_20 corresponds to two discrete cases. SiO_2_@sericin_10 consists of a “patchy” coating of the SiO_2_ surface, while SiO_2_@sericin_20 consists of a complete coating of the SiO_2_ surface by the grafted sericin protein. In the following, the interfacial surface charge properties of the hybrids were studied by dynamic light scattering.

#### 3.1.4 Dynamic Light Scattering (DLS)

##### 3.1.4.1 Size Distribution and ζ Potential Analysis

DLS allows the study of the interfacial dynamics of particles when in contact with the aqueous phase at various pH values. Panels A–C of Figure 5 present the DLS size distribution data for the three materials. We underline that the sizes shown correspond to aggregates formed and/or stabilized in the aquatic dispersions of the materials. SiO_2_ exhibits three distinct size distributions, with average agglomerate sizes of 156 ± 25 nm (major fraction), 669 ± 300 nm, and 1785 ± 630 nm. Interestingly, an aqueous sericin dispersion (Figure 5B) shows a broad aggregate size distribution ranging from 50 to 4000 nm. This broad aggregate size distribution indicates that the service macromolecules form a quasi-continuous aggregate size, i.e., small number of sericin macromolecules form the small aggregates, while progressively larger aggregates are also stabilized, in the same suspension. In contrast to the results described above, the size distributions of the hybrid materials vary significantly. SiO_2_@sericin_20 (Figure 5C) demonstrates one well-defined aggregate size of 550 ± 40 nm, while SiO_2_@sericin_10 (Figure 5D) exhibits two distinct size distributions: one at 210 ± 30 nm and one at 850 ± 60 nm.

**Figure 5:**
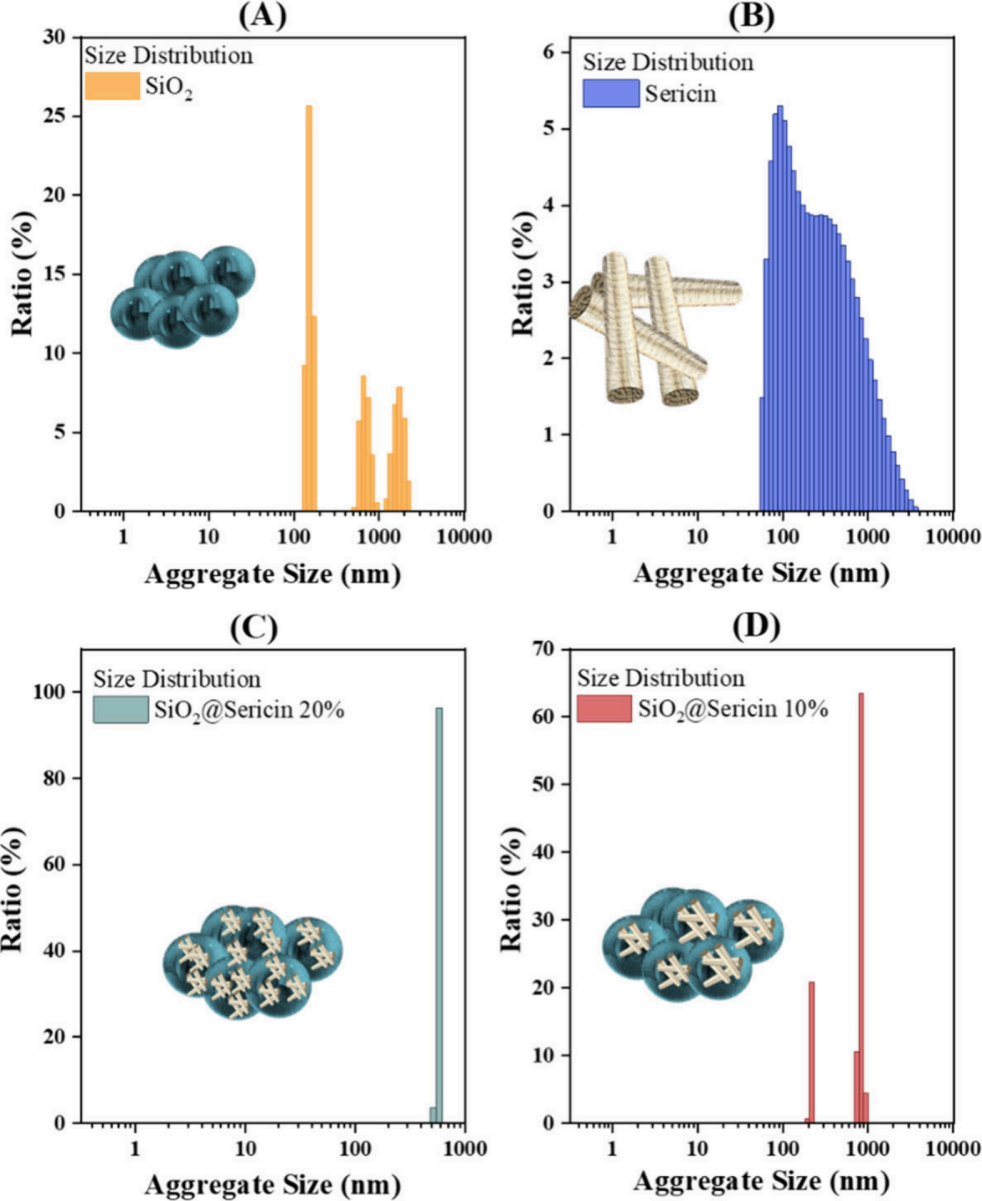
DLS aggregate size distributions for the materials in aqueous suspensions: (A) SiO_2_, (B) solution of sericin, (C) SiO_2_@sericin_20, and (D) SiO_2_@sericin_10.

The present DLS analysis demonstrates that surface grafting of sericin onto SiO_2_ has a decisive impact on the interfacial dynamics of the SiO_2_@sericin aggregates in aqueous solution in two ways. (1) The SiO_2_@sericin_10 and SiO_2_@sericin_20 loadings result in a homogenization of the aggregate size, i.e., versus sericin in solution or the SiO_2_ particles alone. (2) The 20% loading results in a considerably uniform 850 nm aggregate, which is larger than SiO_2_. The 10% loading results in less uniform/smaller aggregates of 200 and 550 nm that are, nevertheless, well-defined.

Overall, the present IR, Raman, BET, and DLS data reveal that surface-grafted sericin plays a dominant role in the aquatic/interfacial dynamics of the SiO_2_@sericin hybrids. The 20% material represents a limiting case where the surface is fully covered with sericin. The surfacial sericin drives the interfacial particle–particle aggregation to a unique, thermodynamically stable formation of 850 nm, i.e., corresponding to ∼10 particles of SiO_2_@sericin_20 in each aggregate.

##### 3.1.4.2 ζ Potential

Panels A and B of Figure 6 present the ζ potential measurements for SiO_2_@sericin_20 and SiO_2_@sericin_10, respectively. For comparison, the ζ data for SiO_2_ and sericin are also included. Typically, the ζ versus pH data represent the interfacial protonation/deprotonation dynamics of the particle when it is in contact with the H_2_O solvent. A positive ζ, which usually prevails at acidic pH, evidences protonated states at the {particle/H_2_O} interface, while a negative ζ, which usually prevails at alkaline pH, evidences deprotonated states at the {particle/H_2_O} interface. The pH where the positive and negative charges surface states are equal determines the point of zero charge (PZC).^64^ SiO_2_ is a unique exception to the ζ theory; i.e., it is the only oxide whose surface does not stabilize positive charges.^64–66^ SiO_2_ at acidic pH shows a ζ ∼ 0, shows a PZC ≈ 3, and attains a negative ζ at alkaline pH (see Figure 6A), in agreement with the literature.^64–66^ This ζ versus pH trend is typical for SiO_2_; i.e., the surface consists of silanol groups (Si–OH),^65,66^ which when pH > PZC ∼ 3 are deprotonated, forming (Si–O^–^) groups that prevail, thus giving negative ZP values, with a max ζ = −27 mV at pH 9 (Figure 6).

**Figure 6:**
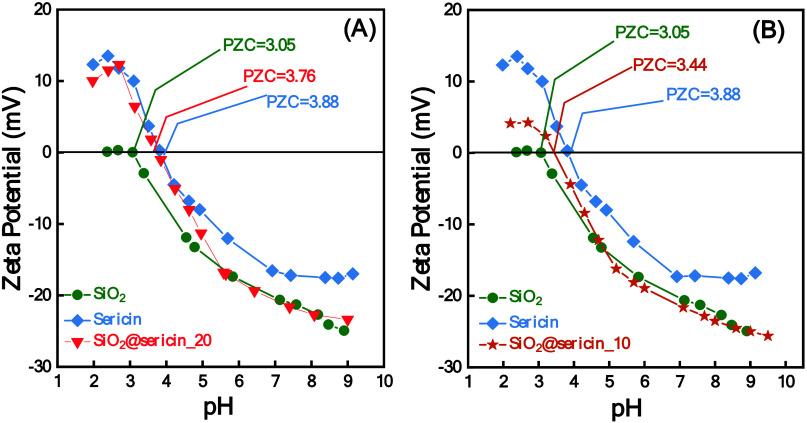
ζ potential as a function of pH for (A) SiO_2_@sericin_20 and (B) SiO_2_@sericin_10. For comparison, the ζ data for SiO_2_ and sericin are also included in each panel.

Sericin shows a distinct ζ versus pH profile with a PZC = 3.88. When pH < PZC, sericin displays positive ζ values due to the protonation of amino (−NH_3_^+^) groups, while when pH > PZC, ζ is negative due to the deprotonation of carboxyl (−COO^–^) and/or phenolic R–OH groups.^67,68^

Interestingly, the ζ versus pH profile for SiO_2_@sericin_20 (Figure 6A) closely resembles that of the aqueous sericin solution (Figure 6A), with the same PZC value and similar ζ values in the acidic range of pH 2–4. In contrast, SiO_2_@sericin_10 (Figure 6B) displays a ζ versus pH profile intermediate between those of sericin and SiO_2_. The data in panels A and B of Figure 6 clearly indicate that in SiO_2_@sericin_20, where the SiO_2_ surface is fully coated by sericin, the ζ versus pH profile is determined by protonation/deprotonation events on sericin, while SiO_2_ plays no role or a secondary role. In SiO_2_@sericin_10, where only part of the SiO_2_ surface is coated by sericin, the ζ versus pH profile is determined by protonation/deprotonation events on both sericin and SiO_2_.

Overall, the present DLS data as well as the physicochemical characterizations mentioned above provide a conclusive structural picture. In SiO_2_@sericin_10, both sericin and the SiO_2_ surface are exposed to the solvent medium. The moieties of sericin and SiO_2_ are both available to participate in interfacial interactions. In contrast, with regard to SiO_2_@sericin_20, the sericin dominates the SiO_2_ surface. The moieties of sericin are the only ones available to participate in interfacial interactions. As we show hereafter, this suggests that the knowledge of key importance is understanding the antioxidant/antiradical dynamics of these materials.

### 3.2 Antioxidant Activity of Hybrid Materials against DPPH Radicals: HAT Process

The DPPH method was employed to investigate the antioxidant activity of sericin and SiO_2_@sericin_10 and SiO_2_@sericin_20. The DPPH method is well established to monitor antioxidant HAT.^45,55,69^ The effectiveness of an antioxidant is indicated by the extent of the decrease in the DPPH-radical concentration, easily monitored by its 515 nm absorbance in solution.^70^ Traces i and ii of Figure 7 illustrate the antioxidant activity of sericin at two concentrations against DPPH radicals. The data presented here reveal that sericin, when isolated from the silk fiber, bears some HAT antioxidant activity. More importantly, the data in traces iii and iv of Figure 7 show that SiO_2_@sericin_10 and SiO_2_@sericin_20 hybrids exhibit better antioxidant activity than sericin alone.

**Figure 7:**
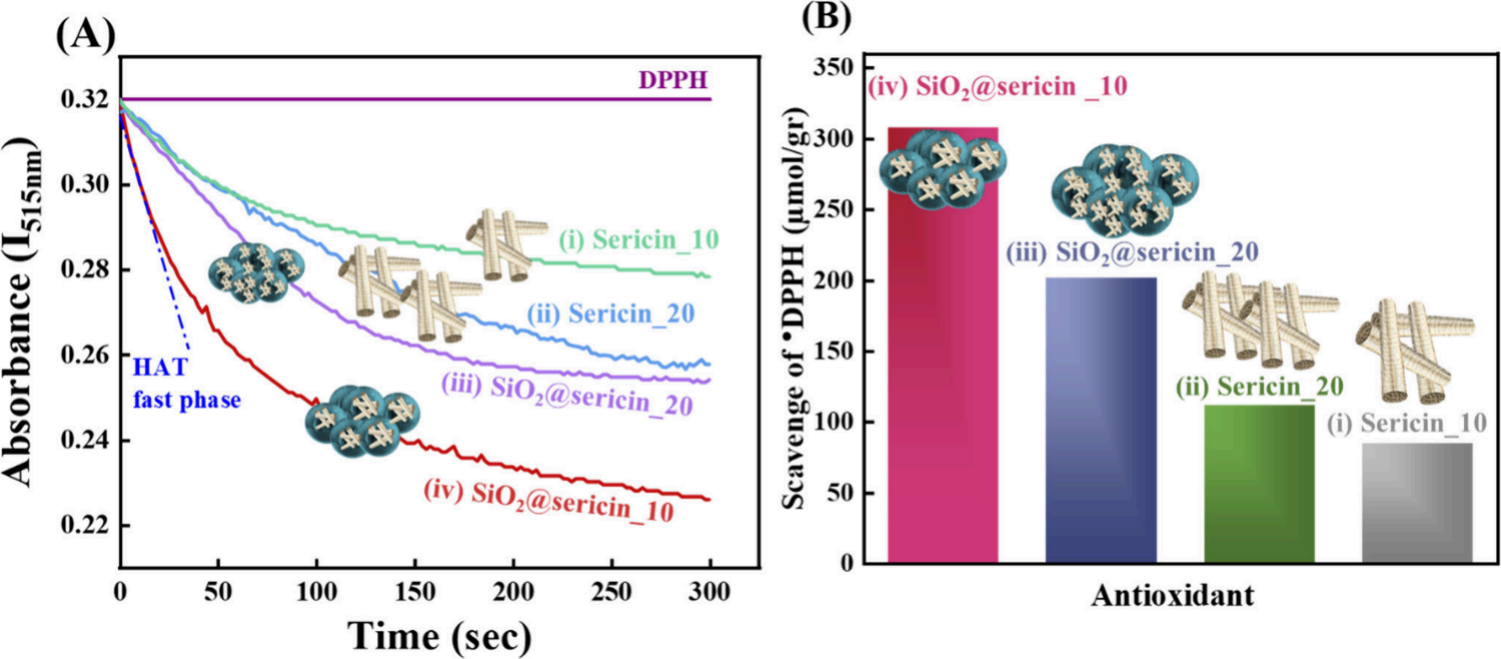
(A) Kinetic analysis of the antioxidant hydrogen atom transfer (HAT) activity of (i) sericin_10, (ii) sericin_20, (iii) SiO_2_@sericin_10, and (iv) SiO_2_@sericin_20 was conducted using the DPPH assay. The onset of the HAT phase is identified by the tangent to the initial decay phase in the reaction curve. (B) Bar graph illustrating the number of moles of DPPH radicals neutralized per gram of sample.

Among the two SiO_2_@sericin hybrids, the data in traces iii and iv of panels A and B of Figure 7 demonstrate that SiO_2_@sericin_10, containing 10% sericin, demonstrates the highest antioxidant activity. Interestingly, in SiO_2_@sericin_20, i.e., with high (20%) sericin content, the HAT antioxidant activity of the hybrid material decreases. According to panels A and B of Figure 7, the materials can be ranked in ascending order of antioxidant activity as follows: SiO_2_@sericin_10 > SiO_2_@sericin_20 > sericin_20 (20% aqueous solution) > sericin_10 (10% aqueous solution).

According to the established method of the HAT process, the initial fast kinetics in Figure 7A (blue dotted line) reflects exclusively the quenching of DPPH radicals via a HAT mechanism,^71^ where one proton and one electron are in tandem transferred simultaneously from the same donor (the antioxidant) to the DPPH acceptor.^72^ Quantitative analysis of the DPPH quench kinetics provides the number of micromoles of DPPH radicals per gram of material (illustrated in Figure 7B and listed also in Table 3). Accordingly, 1 g of SiO_2_@sericin_10 scavenges 308 μmol of DPPH radicals while 1 g of SiO_2_@sericin_20 scavenges 202 μmol of DPPH radicals. Both hybrids show considerably higher DPPH scavenging activity than an aqueous sericin solution, i.e., 20% (w/w) and 10% (w/w) solutions scavenge 112 and 85 μmol of DPPH radicals, respectively (Table 3).

**Table 3:** DPPH-Radical Quenching Capacity (micromoles per gram of antioxidant sample (material or solution))

material	DPPH scavenging (μmol/g of sample) (±3)
sericin (10% (w/w) aqueous solution)	85
sericin (20% (w/w) aqueous solution)	112
SiO_2_@sericin_20	202
SiO_2_@sericin_10	308

The present DPPH antioxidant results reveal that immobilizing sericin onto the silica surface enhances its antioxidant activity versus the aqueous solution of sericin with the same sericin content. In the case of the best performing material, SiO_2_@sericin_10, the enhancement factor is 308/85 > 350%. An analogous, but less dramatic, increase in antioxidant activity is obtained for SiO_2_@sericin_20, where the enhancement ratio versus 20% sericin in solution is 202/112 > 180%. Interestingly, SiO_2_@sericin_10, i.e., the hybrid material with less sericin loading, affords more DPPH scavenging, a trend that is not observed when aqueous sericin solutions are used as an antioxidant source. This evidence shows that the interfacial association of 10% sericin on SiO_2_ is of key importance for the HAT process. As we discussed above, this is related to an optimal disposition of the sericin moieties on the SiO_2_ surface, which allows both the SiO_2_ surface and sericin to partake in the HAT process. This beneficial synergistic role of the SiO_2_ surface with the HAT efficiency of grafted antioxidants has been previously documented for the case of gallic acid@SiO_2_,^43^ hyaluronic components@SiO_2_,^45^ humic polymers@SiO_2_,^46^ ands oligopeptides@SiO_2_.^35^ All of these data, as well as the present data, highlight the beneficial role of the surficial Si–OH groups on HAT = [H^+^/e^–^], via local H-bonding and electrostatic interactions.^44,45^

### 3.3 Assessment of Hydroxyl-Radical Scavenging Activity

To investigate the OH-radical scavenging efficiency, we have used the protocol detailed in Assessment of the Hydroxyl-Radical Scavenging Capacity. In brief, known amounts of OH radicals were generated using the Fenton system (Fe^2+^/H_2_O_2_). The OH radicals were detected with the standard spin-trapping method using DMPO, and the solution containing the stable DMPO–OH radicals displays a characteristic four-line EPR spectrum with a 1:2:2:1 intensity pattern.^50,73–76^ The OH quenching effect of an antioxidant is measured quantitatively by a decrease in the EPR spectra of the DMPO–OH radicals. As a control, we note that in our system the only radicals generated were OH radicals; no additional or any secondary radicals were detected in these experiments.

To assess the OH quenching efficiency of an aqueous sericin solution, varying concentrations of sericin (*C* = 33 ppm, *C* = 67 ppm, and *C* = 133 ppm) were added to the Fenton reaction mixture, 30 s after its initiation, i.e., where OH-radical production reached a steady state. Then, the system was allowed to evolve and/or interact for additional selected time intervals, from seconds to minutes; subsequently, the DMPO spin trap was added, i.e., to map the time kinetics of the OH radicals. Figure 8 shows the EPR spectra of the DMPO–OH radicals, detected at 1 min, generated by the Fenton reaction (Figure 8A,B (i)) or in the presence of an aqueous sericin solution (Figure 8A,B (ii–iv)), where it is demonstrated that the addition of sericin significantly decreases the concentration of DMPO–OH radicals. Specifically, quantitative analysis of the EPR data of traces iii of panels A and B of Figure 8 shows that 1 g of a 10% aqueous solution of sericin scavenges 53 μmol of ^●^OH while 1 g of a 20% aqueous solution of sericin scavenges 70 μmol of ^●^OH (Table 4). This result, rather surprisingly, indicates that the OH scavenging efficiency of sericin is nonlinear versus mass. Lower sericin concentrations are more efficient per mass. We attribute this to the aggregation of sericin at higher concentrations, which inhibits some of the aggregate sites from reacting with the OH radicals.

**Figure 8:**
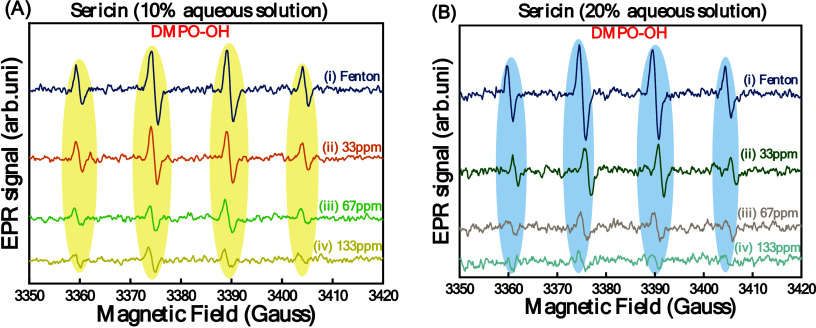
EPR spectra of OH radicals generated by a Fenton reaction (traces i) detected at 1 min. (A) Quenching effect of a 10% aqueous sericin solution. (B) Quenching effect of a 20% aqueous sericin solution. This phenomenon becomes clearer if we examine the data in panels A and B for a similar sericin concentration; e.g., 33 ppm sericin when taken from the 20% stock solution was less efficient than 33 ppm taken from the 10% stock solution.

**Table 4:** Hydroxyl (^●^OH)-Radical Quenching Capacity (micromoles per gram of antioxidant material)

material	OH scavenging (μmol/g of material) (±0.01)
sericin (10% (w/w) aqueous solution)	53
sericin (20% (w/w) aqueous solution)	70
SiO_2_@sericin_20	100
SiO_2_@sericin_10	120

This finding is pertinent to our observation of the lower OH scavenging efficiency for the highly loaded SiO_2_@sericin_20 material versus SiO_2_@sericin_10 (see Figure 9A,B). Specifically, the data show that 1 g of SiO_2_@sericin_10 quenches 120 μmol of ^●^OH (Table 4) versus 100 μmol of ^●^OH quenched per gram of SiO_2_@sericin_20.

**Figure 9:**
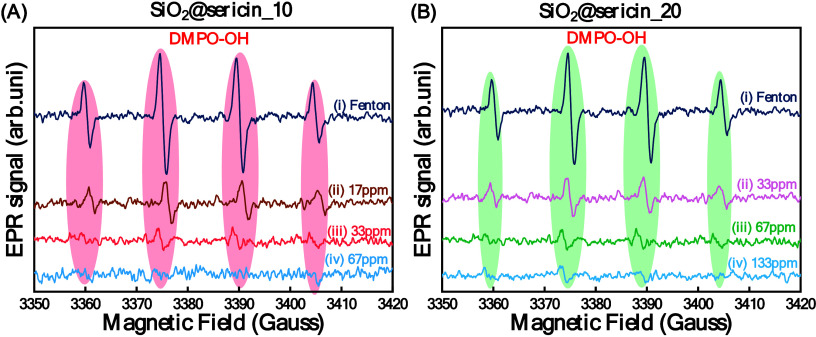
Electron paramagnetic resonance (EPR) spectra of (A) SiO_2_@sericin_10 ((ii) 17 ppm, (iii) 33 ppm, and (iv) 67 ppm) and (B) SiO_2_@sericin_20 ((ii) 33 ppm, (iii) 67 ppm, and (iv) 133 ppm).

All of these data can be visualized and compared in panels A and B of Figure 10 and Table 4. Accordingly, we conclude that when normalized per gram, SiO_2_@sericin_10 exhibits the highest ^●^OH antioxidant activity, immobilization of sericin onto the silica surface enhances the material’s capacity to scavenge ^●^OH radicals, and increasing the amount of sericin, either on the silica surface or in aqueous solution, results in decreased ^●^OH antioxidant activity.

**Figure 10:**
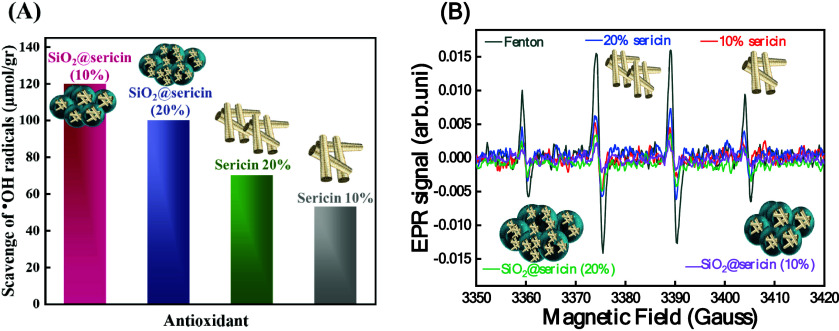
(A) Micromoles of hydroxyl (^●^OH) radicals scavenged per gram of material and (B) electron paramagnetic resonance (EPR) spectra for all materials.

### 3.4 Comparative Analysis of the Antioxidant Capacity of Materials against ^●^OH and DPPH Radicals

Table 5 presents a summary of the present data on the antioxidant capacities versus ^●^OH and DPPH radicals for the materials studied herein, and Figure 11 presents a double-Y presentation of the data.

**Table 5:** DPPH- and Hydroxyl-Radical Quenching Capacity (micromoles per gram of antioxidant)

material	antioxidant HAT efficiency (μmol of DPPH/g of sample) (±0.1)	OH scavenging (μmol/g of sample) (±0.01)
aqueous solution of sericin (10% (w/w))	85	53
aqueous solution of sericin (20% (w/w))	112	70
SiO_2_@sericin_10	308	120
SiO_2_@sericin_20	202	100

**Figure 11:**
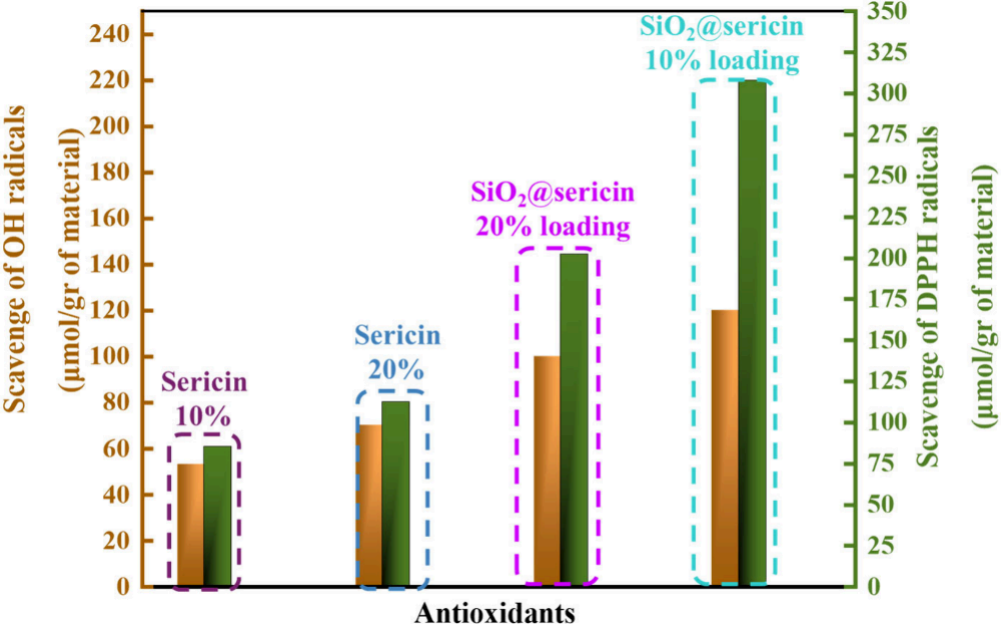
Scavenging capacity for hydroxyl (^●^OH) (left *Y*-axis) and DPPH (right *Y*-axis) radicals, expressed in micromoles per gram of each antioxidant material.

The data in Figure 11 clearly reveal the superiority of the SiO_2_@sericin_10 hybrid versus all other materials, whose antioxidant activity clearly comes from sericin, since neither chloropropyl nor silica exhibits any antioxidant activity^44,45^ based on control experiments that were conducted (see Figure S2). Upon comparison of SiO_2_@sericin_10 versus its SiO_2_@sericin_20 counterpart, the SiO_2_@sericin_10 hybrid is by 180% more efficient in the DPPH (HAT) process and by >20% more efficient in ^●^OH quenching. This comparison reveals that the relative trends versus DPPH and OH radicals are different, which stems from fundamentally different mechanisms of their quenching processes. As we have disused in detail recently,^44,45,49,54^ DPPH quenching is a HAT process mainly occurring via hydroxyl groups, most probably through the OH groups of the serine residue being the most abundant amino acid in sericin herein (∼30%),^17^ while ^●^OH quenching is a more complex radical mechanism that involves either a SET mechanism from a phenolic OH or HAT from a suitable C–H site. The possibility that the observed antioxidant activity can be attributed to some extent to cysteine residues^77^ is unlikely since its abundance in sericin is minimal (∼1%).^17^

Moreover, the data reveal that the immobilization of sericin onto the silica surface enhances its antioxidant activity. For example, when sericin is immobilized on the silica surface at a 10% loading, the DPPH scavenging radicals increased by ∼350% versus the 10% aqueous sericin solution (Figure 11). Similarly, with regard to the ^●^OH antioxidant capacity, immobilizing sericin on the silica surface at a 20% loading increases the activity of the hybrid material by ∼140% compared to that with a 20% aqueous sericin solution, while immobilizing sericin at a 10% loading results in an ∼230% increase in antioxidant activity versus a 10% aqueous sericin solution.

Interestingly, as the amount of immobilized sericin on the silica surface increases, there is a corresponding decrease in the capacity to scavenge DPPH and hydroxyl radicals (Figure 11). As we discussed based on the IR, Raman, BET, and DLS data, this stems from the interfacial topography of the sericin on the SiO_2_ surface. At the optimal 10% loading, the maximum fractions of antioxidant moieties of the sericin macromolecule are active and/or accessible toward radical quenching. In contrast, at the high/saturating 20% loading, the macromolecular configuration turns out to a less active/more aggregated configuration. However, this trend is not observed in aqueous solutions of sericin since we expect the steric hindrance induced by the high concentration to be less severe in the liquid phase.

## 4 Conclusion

This study presents the successful design and synthesis of a new class of hybrid antioxidant materials through the covalent grafting of sericin, a silk-derived protein, onto silica (SiO_2_) nanoparticles. Two hybrid variants, SiO_2_@sericin_10 and SiO_2_@sericin_20, were systematically evaluated to investigate their impact of sericin loading on the antioxidant performance. The findings clearly demonstrate that covalent immobilization of sericin onto the SiO_2_ surface significantly enhances its radical scavenging capacity compared to sericin in an aqueous solution alone. Among the two formulations, the SiO_2_@sericin_10 hybrid exhibited superior antioxidant activity, effectively neutralizing 308 μmol of DPPH radicals and 120 μmol of hydroxyl radicals per gram. This performance is notably higher than those of both SiO_2_@sericin_20 and unbound sericin, highlighting the critical influence of surface coverage and molecular accessibility. Characterization measurements support the conclusion that the sericin conformation on the SiO_2_ surface plays a decisive role. At the optimized 10% loading, sericin adopts a more dispersed and accessible configuration, maximizing the exposure of its antioxidant moieties. Conversely, the denser conformal coating observed at 20% loading imposes steric hindrance, reducing the effectiveness of radical quenching. Overall, this work underscores the importance of interfacial engineering in hybrid material design and provides a clear pathway for enhancing the functional performance of bioorganic–inorganic composites. The approach of covalently tethering antioxidant-rich biomolecules like sericin onto inorganic carriers such as SiO_2_ offers a promising platform for developing advanced antioxidant systems applicable in biomedicine, cosmetics, and environmental remediation.

## Supplementary Material



## Notes

Pore size distribution and analysis of SiO_2_ and SiO_2_@sericin hybrids with 10% and 20% sericin loadings using BET (Figure S1) and hydroxyl-radical scavenging activity of SiO_2_ and SiO_2_@Cl materials (Figure S2) (PDF)
