# Supplementary material for: Antioxidant Activity of SiO2@{Sericin} Hybrids: A Comparable OH-Radical and DPPH-Radical Scavenging Study
Source: Langmuir. 2025 Sep 24;41(39):26715–29. doi: 10.1021/acs.langmuir.5c03085 (PMC12509319; doi:10.1021/acs.langmuir.5c03085)
Supplement: Supplementary file 1 [file la5c03085_si_001.pdf]

Supporting Information

Antioxidant Activity of SiO_2@{Sericin} Hybrids: A Comparable OH-Radical and DPPH-Radical Scavenging Study

Annita Theofanous[a], *George Theofilou*[b], *Yiannis Deligiannakis*[b], *Maria Louloudi*[a*]

[a] Laboratory of Biomimetic Catalysis & Hybrid Materials, Department of Chemistry

University of Ioannina, GR-45110 Panepistimioupoli Ioannina, Greece

[b] Laboratory of Physical Chemistry of Materials & Environment, Department of Physics

University of Ioannina, GR-45110 Panepistimioupoli Ioannina, Greece

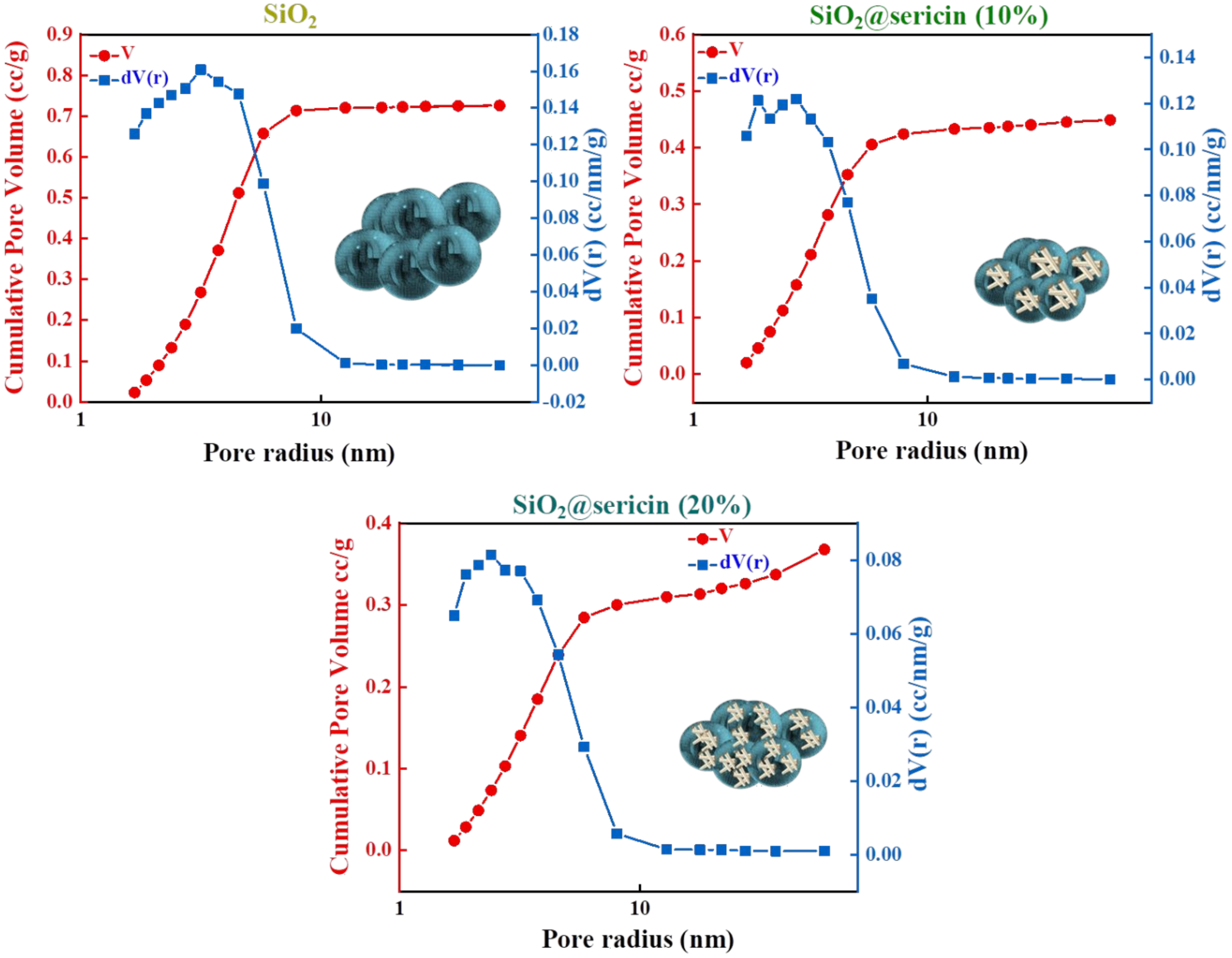

Figure S1. Pore-size analysis for the materials SiO_2 (up and left), SiO_2@sericin (10%) (up and right), and SiO_2@sericin (20%) (down, middle).

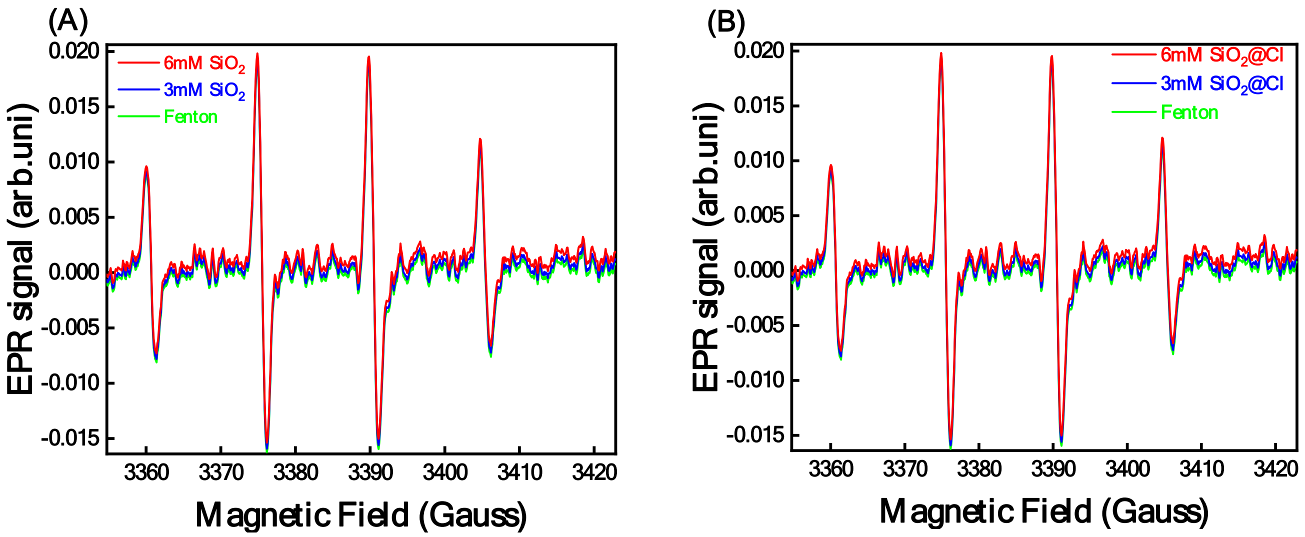

Figure S2. Comparison of (A) SiO_2 and (B) SiO2@Cl materials in their scavenging of hydroxyl radicals (•OH).
